# Association between primary tumor location and lymph node metastatic patterns in gastric cancer: A single-center retrospective study

**DOI:** 10.1097/MD.0000000000048232

**Published:** 2026-04-10

**Authors:** Zhiqiang Dong, Liping Fu, Long Yang, Benxin Yang, Shuli Song, Jiangqiao Zhao

**Affiliations:** a Department of Hepatobiliary and Pancreatic Surgery, Cangzhou People’s Hospital, Cangzhou City, Hebei Province, China; b Interventional Radiongraphy, Cangzhou People’s Hospital, Cangzhou City, Hebei Province, China; c Department of Gastroenterology, Cangzhou People’s Hospital, Cangzhou City, Hebei Province, China.

**Keywords:** gastric cancer, LNR, lymph node metastasis, primary tumor location, prognosis

## Abstract

Gastric cancer is a common malignancy of the digestive system worldwide, and lymph node metastasis is a key determinant for prognosis assessment and therapeutic decision-making. Patterns of lymphatic drainage and nodal spread may differ according to the primary tumor location. This study aimed to investigate the relationship between primary tumor site and lymph node metastatic characteristics. A total of 160 patients who underwent curative gastrectomy between February 2022 and June 2024 were retrospectively reviewed. According to the primary tumor location, patients were categorized into the cardia, body, and antrum groups. Lymph node metastasis rate, involved stations, metastatic extent, and lymph node ratio (LNR) were compared among groups. Multivariate logistic regression was performed to identify independent risk factors. The metastasis rates in the cardia, body, and antrum groups were 74.5%, 63.5%, and 58.5%, respectively, showing a decreasing trend. Cardia tumors predominantly involved stations No. 1 and No. 2, whereas antral tumors mainly involved stations No. 5 and No. 6 (*P* < .05). Patients with poorly differentiated or special-type histology had significantly higher LNRs. Multivariate analysis indicated that primary tumor located in the cardia, T3–T4 stage, and poor differentiation were independent risk factors (*P* < .05). Patients with diffuse-type lymphatic spread had markedly lower 3-year disease-free and overall survival compared with those with localized spread. Primary tumor location is closely associated with lymph node metastatic patterns in gastric cancer. Cardia tumors are more prone to longitudinal and cross-regional metastasis, while high T-stage and poor differentiation are linked with extensive nodal involvement and worse prognosis. Combined assessment of primary site and LNR may provide a useful reference for preoperative evaluation and lymphadenectomy planning.

## 1. Introduction

Gastric cancer remains one of the most prevalent malignancies of the digestive tract, with persistently high incidence and mortality in many regions worldwide. Global cancer statistics indicate that it accounts for a considerable proportion of new cancer diagnoses and cancer-related deaths, particularly in East Asian countries. Lymph node metastasis is a critical determinant for postoperative prognosis, staging, surgical planning, and adjuvant strategies. Extensive evidence has demonstrated strong associations between the degree of nodal involvement – such as the number of positive nodes, lymph node ratio (LNR), and the extent of metastatic stations – and overall survival (OS) as well as recurrence risk in patients with gastric cancer.^[1]^

In curative gastrectomy, systematic lymphadenectomy (e.g., D1, D1+, D2 dissection) is regarded as a pivotal component for achieving adequate local control and improving long-term outcomes.^[2]^ Accurate assessment of the risk and pattern of nodal metastasis before or during surgery is therefore essential for optimizing the extent of dissection, minimizing residual disease, and guiding postoperative treatment planning.^[3]^ Importantly, lymph node metastasis should not only be interpreted as a binary event but also evaluated in terms of metastatic stations, anatomical distribution (localized vs cross-regional), and the proportional burden reflected by LNR – dimensions increasingly emphasized in recent research.^[4]^

The primary tumor location – cardia, body, or antrum – may be closely related to its lymphatic drainage pathways and subsequent nodal spread. Based on the Japanese Gastric Cancer Association (JGCA) classification, gastric lymph nodes are organized into stations No. 1 to No. 16, and several studies have observed distinct metastatic tendencies among tumors arising from different anatomical sites.^[5]^ For instance, Li et al reported that proximal tumors exhibit higher nodal involvement rates and characteristic metastatic station patterns.^[6]^ Thus, incorporating primary tumor location into preoperative prediction models may enhance the precision of lymphadenectomy planning.

From a pathological perspective, factors such as tumor invasion depth (T stage), histological differentiation, tumor size, and morphological subtype (e.g., signet-ring cell carcinoma, mucinous adenocarcinoma) have been confirmed to correlate with the likelihood and burden of nodal spread, as well as the number of affected stations.^[7-9]^ A systematic review indicated that although lymph node metastasis is relatively uncommon in T1 gastric cancer, it still occurs and escalates significantly as tumors penetrate deeper layers toward T4.^[10]^ Poorly differentiated or special histological types often show a stronger propensity for infiltration and dissemination, resulting in markedly elevated LNR values.^[11]^

Regarding preoperative evaluation, imaging modalities (contrast-enhanced CT/MRI), endoscopic assessment, and serum tumor markers (CEA, CA19-9, CA72-4) may provide indications of potential nodal involvement but remain limited in diagnostic accuracy.^[12]^ Therefore, integrating tumor location with pathological and clinical variables may offer an alternative approach to predicting lymphatic spread patterns, supporting risk stratification and individualized surgical decision-making.

Recent advances have further revealed that lymph node metastasis in gastric cancer is a complex, multistep biological process rather than a simple proximal-to-distal extension. Interactions among tumor cells, lymphatic vessels, immune microenvironment, and molecular mechanisms play distinct roles. For example, Zuo et al demonstrated that metastatic lymph nodes may exhibit clonal evolutionary trajectories different from the primary tumor, underscoring their unique biological significance.^[7]^ Additionally, the occurrence of “skip metastasis,” in which distal nodes are involved despite negative regional nodes, poses new challenges to determining the optimal extent of lymphadenectomy.^[13]^

Against this background, systematically elucidating the associations between primary tumor location and lymph node metastatic patterns – including metastasis rate, involved stations, LNR, and localized versus cross-regional spread – has important clinical implications for preoperative evaluation, lymphadenectomy mapping, individualized surgical strategy, and postoperative management. Therefore, based on a single-center retrospective cohort, we analyzed patients who underwent curative gastrectomy with systematic lymph node dissection between February 2022 and June 2024 to clarify how primary tumor location influences lymphatic metastatic behavior, aiming to provide practical guidance for surgical decision-making.

## 2. Materials and methods

### 2.1. Study design and ethical approval

This study was approved by the Ethics Committee of Cangzhou People’s Hospital. This single-center retrospective observational study was conducted using clinical data from patients who underwent curative gastrectomy at our gastrointestinal surgery department between February 2022 and June 2024. The aim was to clarify the association between primary tumor location and lymph node metastatic patterns, thereby informing preoperative staging, lymphadenectomy planning, and individualized treatment strategies. The study protocol was reviewed and approved by the institutional ethics committee. Owing to the retrospective nature of the analysis, the requirement for informed consent was waived.

### 2.2. Patient selection and grouping criteria

A total of 160 patients with pathologically confirmed primary gastric adenocarcinoma were included. All individuals underwent radical gastrectomy with systematic lymphadenectomy. Inclusion criteria were: postoperative histopathological confirmation of primary gastric adenocarcinoma; D1+ or D2 lymph node dissection performed; complete clinical and imaging records; absence of distant metastasis (M0); and age ≥ 18 years.

Exclusion criteria were: history of other primary malignancies or previous gastric cancer; preoperative chemotherapy, radiotherapy, targeted therapy, or immunotherapy; palliative or exploratory surgery; and incomplete pathological or follow-up data. Patients were categorized into 3 groups based on primary tumor location: cardia (proximal), body (middle), and antrum (distal). According to metastatic patterns, lymph node involvement was further classified as localized (confined to nodes corresponding to the primary region) or disseminated (cross-regional or distal nodal spread).

In accordance with international gastric cancer surgery guidelines, lymph node retrieval aimed to include at least 16 nodes to ensure adequate pathological staging.

### 2.3. Data collection and variable definitions

Two independent investigators retrieved and verified clinical information from the hospital electronic medical record (hospital information system), imaging archive (picture archiving and communication system), and pathology database. A third senior reviewer resolved any discrepancies. Extracted data included demographic and clinical characteristics, preoperative tests, surgical records, pathological findings, molecular markers, and follow-up outcomes. Baseline variables comprised sex, age, body mass index (BMI), body surface area, smoking and alcohol history, comorbidities (hypertension, diabetes, coronary artery disease, chronic liver disease, etc), presenting symptoms, American Society of Anesthesiologists classification, nutritional risk screening (NRS-2002), and serum albumin.

Preoperative examinations included endoscopic findings (tumor location, circumferential involvement, morphological type), contrast-enhanced CT/MRI (maximum tumor diameter, depth of invasion, number of suspicious nodes, proportion of nodes with short axis > 8 mm), tumor markers (CEA, CA19-9, CA72-4, α-fetoprotein), and standard laboratory tests.

Surgical and pathological variables included operative procedure (distal, proximal, or total gastrectomy), surgical approach (open, laparoscopic, or robotic), extent of lymphadenectomy (D1+ or D2), operative time, intraoperative blood loss, margin status (R0/R1 [R0 = resection margin negative/R1 = resection margin positive]), tumor location (distance from cardia or pylorus), tumor size, histological subtype (well/moderately/poorly differentiated adenocarcinoma, signet-ring cell carcinoma, mucinous adenocarcinoma), depth of invasion (T1–T4), perineural and vascular invasion, serosal involvement, total number of harvested lymph nodes, and number of positive nodes.

All cases were staged according to the eighth edition AJCC system.

For patients with available data, molecular and immunohistochemical markers, including human epidermal growth factor receptor 2 (immunohistochemistry/fluorescence in situ hybridization), Ki-67, p53, E-cadherin, vascular endothelial growth factor, matrix metalloproteinase-9, microsatellite instability status, Epstein–Barr virus infection, and CD8+ T-cell infiltration, were recorded. Follow-up variables – recurrence or metastasis at 6, 12, 24, and 36 months, disease-free survival (DFS), and OS – were included when available. All data were anonymized and underwent double entry and consistency validation to ensure accuracy.

### 2.4. Study endpoints and analytical framework

The primary objective was to evaluate the relationship between tumor location and lymph node metastatic patterns from clinical, pathological, and biological perspectives.

First, baseline characteristics – such as demographic factors, BMI, comorbidity profile, tumor size, differentiation, nutritional parameters, and biochemical indices – were compared among the 3 tumor-location groups.

Second, overall lymph node metastasis rates were calculated for each location to assess anatomical variability in metastatic propensity. Lymph node station involvement was recorded according to the fifth edition of the Japanese Gastric Cancer Association (JGCA) classification (No. 1–No. 16). Distinct metastatic pathways were analyzed, such as No. 1/No. 2/No. 7 involvement in cardia tumors, No. 3/No. 4/No. 9 in body tumors, and No. 3/No. 5/No. 6 in antral tumors.

Third, differences in metastatic extent and positive node number were evaluated across T stages (T1–T4) to explore the relationship between longitudinal invasion depth and horizontal lymphatic dissemination.

Fourth, lymph node involvement, LNR, and cross-regional metastasis were compared across histological grades and special subtypes to elucidate the effect of differentiation on tumor aggressiveness. Lymph node ratio (positive nodes/total nodes retrieved) was calculated for all cases and stratified into <0.1, 0.1 to 0.3, and >0.3 groups.

Fifth, nodal burden and distribution were compared across AJCC stages I–III, identifying trends in metastatic spread with disease progression.

Multivariate logistic regression was applied, using involvement of specific stations (No. 6, No. 9, No. 11, No. 12) as dependent variables and primary tumor location as the independent variable, adjusting for age, sex, T stage, differentiation, and surgical type. Odds ratios (ORs) and 95% confidence intervals (CIs) were calculated to determine whether tumor location independently predicted metastasis to specific nodal stations.

Preoperative serum tumor markers (CEA, CA19-9, CA72-4) were analyzed for their correlations with positive node count, LNR, and metastatic extent, assessing their predictive value for nodal involvement.

For patients with complete follow-up, recurrence rates, DFS, and OS were compared between localized and disseminated metastatic patterns. Subgroup analyses incorporated tumor stage and differentiation to evaluate their prognostic impact and inform postoperative risk stratification.

### 2.5. Statistical analysis

Statistical analyses were performed using IBM SPSS Statistics, version 26.0 (IBM Corp., Armonk). Normality of continuous variables was evaluated with the Shapiro–Wilk test. Normally distributed variables were expressed as mean ± standard deviation and compared using independent-sample *t* tests or 1-way analysis of variance. Non-normally distributed variables were analyzed using the Mann–Whitney *U* test. Categorical variables were presented as counts and percentages and compared using the χ^2^ test or Fisher exact test. Correlations were assessed with Pearson or Spearman methods as appropriate. Multivariate logistic regression was conducted to identify independent predictors of lymph node involvement, expressed as ORs with 95% CIs. For survival analyses, the Kaplan–Meier method was used, and differences between groups were examined with the log-rank test. A 2-sided *P* value < .05 was considered statistically significant.

## 3. Results

### 3.1. Baseline characteristics of the study population

A total of 160 patients were included: 55 with cardia tumors, 52 with tumors in the gastric body, and 53 with antral tumors. No significant differences were observed among the 3 groups regarding age, sex distribution, BMI, smoking or alcohol history, major comorbidities such as hypertension and diabetes, or preoperative nutritional status (all *P* > .05), indicating good comparability across groups. Serum albumin levels and CEA values were also similar among the groups (*P* = .664 and *P* = .627, respectively). Detailed characteristics are shown in Table 1.

**Table 1:** Comparison of baseline characteristics of patients.

Variable	Cardia group (n = 55)	Body group (n = 52)	Antrum group (n = 53)	*t*/χ^2^	*P* value
Age (yr, *x̄* ± *s*)	61.4 ± 10.2	59.8 ± 9.7	58.9 ± 10.9	1.14	.322
Sex (male/female)	38/17	33/19	36/17	0.29	.865
BMI (kg/m^2^, *x̄* ± *s*)	23.4 ± 2.5	23.1 ± 2.7	22.9 ± 2.6	0.45	.636
Smoking history, n (%)	21 (38.2%)	18 (34.6%)	19 (35.8%)	0.13	.936
Alcohol history, n (%)	16 (29.1%)	14 (26.9%)	13 (24.5%)	0.24	.887
Diabetes, n (%)	7 (12.7%)	8 (15.4%)	9 (17.0%)	0.42	.811
Hypertension, n (%)	15 (27.3%)	13 (25.0%)	12 (22.6%)	0.21	.902
Serum albumin (g/L, *x̄* ± *s*)	39.5 ± 3.8	40.1 ± 4.2	39.8 ± 3.9	0.41	.664
CEA (ng/mL, *x̄* ± s)	5.62 ± 3.81	4.97 ± 3.44	5.10 ± 3.57	0.47	.627

BMI = body mass index, CEA = carcinoembryonic antigen.

### 3.2. Lymph node metastasis rates by primary tumor location

The overall lymph node metastasis rates were 74.5% in cardia tumors, 63.5% in tumors of the gastric body, and 58.5% in antral tumors. Although the differences did not reach statistical significance (χ^2^ = 3.62, *P* = .164), a descending trend was evident. Patients with cardia tumors showed the highest metastatic rate, suggesting a greater propensity for upward or mediastinal spread in proximal gastric cancer, whereas antral tumors tended to remain confined to lower gastric regions (Table 2).

**Table 2:** Comparison of lymph node metastasis rates by primary tumor location.

Primary location	Cases (n)	Metastatic cases (n)	Metastasis rate (%)	χ^2^	*P* value
Cardia	55	41	74.5	3.62	.164
Body	52	33	63.5		
Antrum	53	31	58.5		

### 3.3. Distribution patterns of metastatic lymph nodes

Distinct lymphatic distribution patterns were observed among different tumor locations. Cardia tumors most frequently involved stations No. 1 (right cardia) and No. 2 (left cardia), accounting for 49.1% and 43.6%, respectively. Tumors of the gastric body primarily metastasized to stations No. 3 (lesser curvature) and No. 4 (greater curvature), while antral tumors showed predominant involvement of No. 5 (suprapyloric) and No. 6 (infrapyloric) nodes. All differences were statistically significant (*P* < .05). Details are listed in Table 3.

**Table 3:** Distribution of lymph node metastasis by tumor location.

Lymph node station	Cardia (n = 55)	Body (n = 52)	Antrum (n = 53)	χ^2^	*P* value
No. 1 (right cardia)	27 (49.1%)	9 (17.3%)	6 (11.3%)	18.45	<.001
No. 2 (left cardia)	24 (43.6%)	8 (15.4%)	5 (9.4%)	16.21	<.001
No. 3 (lesser curvature)	32 (58.2%)	34 (65.4%)	39 (73.6%)	2.47	.291
No. 4 (greater curvature)	19 (34.5%)	28 (53.8%)	31 (58.5%)	6.72	.035
No. 5 (suprapyloric)	5 (9.1%)	8 (15.4%)	18 (34.0%)	10.84	.004
No. 6 (infrapyloric)	3 (5.5%)	10 (19.2%)	22 (41.5%)	21.13	<.001
No. 9 (around celiac artery)	8 (14.5%)	14 (26.9%)	10 (18.9%)	2.35	.309

### 3.4. Lymph node involvement across T stages

A clear escalation in both metastasis rates and positive node counts was observed with increasing depth of tumor invasion. The metastasis rates were 29.2% in T1, 52.6% in T2, 74.1% in T3, and 86.4% in T4 (*P* < .001). The mean number of positive lymph nodes rose markedly from 2.1 in T1 to 11.5 in T4 disease. Corresponding results are summarized in Table 4.

**Table 4:** Lymph node metastasis according to T stage.

T stage	Cases (n)	Metastatic cases (n)	Metastasis rate (%)	Positive nodes (*x̄* ± *s*)	χ^2^/*t*	*P* value
T1	24	7	29.2	2.1 ± 1.3	–	–
T2	38	20	52.6	4.6 ± 2.7	–	–
T3	54	40	74.1	8.2 ± 3.9	–	–
T4	44	38	86.4	11.5 ± 4.3	4.78	<.001

### 3.5. Metastatic patterns by tumor differentiation

Poorer differentiation was associated with more aggressivenodal spread. The metastasis rate was 52.8% in well-differentiatedadenocarcinoma, 77.8% in poorly differentiated tumors, and as high as 88.2% in special histological types such as signet-ring cell and mucinous carcinoma (*P* < .01). Lymph node ratio also increased with decreasing differentiation (0.18 ± 0.09 in well-differentiated vs 0.33 ± 0.14 in poorly differentiated tumors). Table 5 provides detailed comparisons.

**Table 5:** Lymph node metastatic characteristics by tumor differentiation.

Differentiation	Cases (n)	Metastatic cases (n)	Metastasis rate (%)	LNR (*x̄* ± *s*)	χ^2^/*t*	*P* value
Well differentiated	36	19	52.8	0.18 ± 0.09	–	–
Moderately differentiated	62	40	64.5	0.25 ± 0.11	–	–
Poorly differentiated	45	35	77.8	0.33 ± 0.14	7.86	.001
Special types (signet-ring/mucinous)	17	15	88.2	0.41 ± 0.13	4.52	.002

LNR= lymph node ratio.

### 3.6. Extent of lymphatic spread across tumor, node, metastasis (TNM) stages

Advancing TNM stage was accompanied by markedly wider lymphatic involvement. Stage I patients had a mean of 1.6 involved stations, whereas stage III patients showed involvement of an average of 6.1 stations. The proportion of cross-regional metastasis increased from 10.7% to 55.3% (*P* < .001). These findings suggest a strong positive correlation between disease stage and metastatic extent (Table 6).

**Table 6:** Lymph node metastasis distribution by TNM stage.

TNM stage	Cases (n)	No. of metastatic stations (*x̄* ± *s*)	Cross-regional metastasis (%)	χ^2^/*t*	*P* value
Stage I	28	1.6 ± 0.8	10.7	–	–
Stage II	56	3.4 ± 1.7	32.1	–	–
Stage III	76	6.1 ± 2.4	55.3	8.25	<.001

TNM = tumor, node, metastasis.

### 3.7. Multivariate logistic regression analysis

Multivariate analysis identified primary tumor location – specifically cardia tumors – as an independent predictor of metastasis to specific lymph node stations (OR = 2.62, *P* = .023). Advanced T stage (T3–T4) and poor differentiation were also significantly associated with nodal involvement (*P* < .05). The extent of lymphadenectomy did not significantly influence metastatic risk. Detailed results are presented in Table 7.

**Table 7:** Multivariate logistic regression analysis of predictors for specific lymph node metastasis.

Variable	β	SE	Wald	OR (95% CI)	*P* value
Primary location (cardia vs others)	0.96	0.42	5.22	2.62 (1.18–5.82)	.023
T stage (T3–T4 vs T1–T2)	1.18	0.39	9.15	3.25 (1.54–6.83)	.004
Differentiation (poor/special vs well)	0.83	0.37	5.05	2.30 (1.12–4.71)	.028
Elevated CEA (≥5 ng/mL)	0.71	0.33	4.58	2.03 (1.06–3.89)	.038
Lymphadenectomy extent (D2 vs D1+)	–0.52	0.4	1.69	0.59 (0.27–1.31)	.194

CEA = carcinoembryonic antigen, CI= confidence interval, OR = odds ratio, SE = standard error.

### 3.8. Prognostic comparison of metastatic patterns

Among the 120 patients with follow-up data, 66 exhibited localized nodal metastasis and 54 showed disseminated spread. Patients with disseminated metastasis had significantly higher recurrence rates (44.4% vs 18.2%, *P* = .002) and poorer 3-year disease-free and OS (55.6% and 60.1% vs 78.8% and 84.8%, respectively). These findings indicate a strong association between metastasis pattern and long-term prognosis (Table 8).

**Table 8:** Prognostic comparison between different metastatic patterns.

Metastatic pattern	Cases (n)	Recurrence rate (%)	3-yr DFS (%)	3-yr OS (%)	χ^2^	*P* value
Localized pattern	66	18.2	78.8	84.8	–	–
Disseminated pattern	54	44.4	55.6	60.1	9.43	.002

DFS = disease-free survival, OS = overall survival.

## 4. Discussion

In this retrospective study of 160 patients who underwent curative gastrectomy with systematic lymphadenectomy, we examined how primary tumor location – cardia, body, or antrum – correlates with lymph node metastatic patterns. Although the overall metastasis rates were not statistically different among locations, clear distinctions were observed in the distribution of involved nodal stations. Moreover, T stage, histological differentiation, and metastatic pattern (localized vs disseminated) were all strongly associated with patient prognosis. The present study provides several novel contributions compared with previous research. First, instead of evaluating lymph node metastasis using a single parameter, we integrated multiple dimensions – including primary tumor location, lymph node station distribution, LNR, and metastatic pattern – to construct a more comprehensive analytical framework. Second, by classifying nodal involvement into localized and disseminated spread, we were able to demonstrate a clear association between metastatic pattern and long-term prognosis. Third, the present cohort represents real-world surgical data from a high-incidence region of gastric cancer in China, thereby providing clinically relevant evidence for lymphadenectomy planning in routine clinical practice.

### 4.1. Primary tumor location and nodal station involvement

Our findings indicate that tumors arising in the cardia tended to metastasize to proximal stations such as No. 1 and No. 2, whereas antral tumors more frequently involved No. 5 and No. 6. These results are consistent with previous literature. de Jongh et al^[14]^ reported that proximal gastric cancers typically metastasize to stations 1, 2, 7, and 9, while distal tumors often involve stations 5, 6, and 8. Brisinda et al^[4]^ similarly showed characteristic patterns of nodal spread in distal tumors undergoing D2 lymphadenectomy, supporting anatomical distinctions in lymphatic drainage. Akiyama’s analysis within the Japanese Clinical Oncology Group also confirmed that longitudinal tumor location significantly affects nodal involvement in early gastric cancer.^[15]^ Collectively, these findings underscore the importance of incorporating tumor location into preoperative imaging interpretation and surgical planning to anticipate potential nodal involvement.

### 4.2. Lymph node metastasis rate and extent

In this cohort, cardia tumors demonstrated the highest overall metastasis rate (74.5%), followed by tumors of the gastric body (63.5%) and antrum (58.5%). This proximal-to-distal decreasing trend parallels observations from Khalayleh et al,^[16]^ who identified primary tumor location as an independent determinant of metastasis risk in a large JAMA Network Open cohort. Consistent with our findings, Ma et al^[17]^ reported that increasing T stage was accompanied by a broader nodal metastatic field and higher cross-regional spread. Taken together, both primary tumor site and local invasion depth contribute to determining the scope and complexity of nodal dissemination.

### 4.3. Clinical implications of tumor differentiation and LNR

We observed significantly higher metastasis rates and LNR values in poorly differentiated tumors and special histological subtypes, including signet-ring cell carcinoma and mucinous adenocarcinoma. According to Rosa et al,^[5]^ low-grade tumors exhibit greater lymphatic invasion and heightened propensity for distant spread. The LNR – the proportion of metastatic nodes among all retrieved nodes – has been recognized as a more sensitive marker of tumor burden and lymphadenectomy adequacy than the conventional N classification.^[18]^ Our results further support the prognostic relevance of LNR in postoperative risk stratification.

### 4.4. Skip metastasis

Skip metastasis, defined as involvement of distant nodal stations without proximal nodal disease, is a well-documented phenomenon. Choi et al^[19]^ reported a prevalence of approximately 8% to 15%, attributing its occurrence to aberrant lymphatic pathways, micrometastasis, or insufficient nodal clearance. Zhao et al^[20]^ demonstrated that although skip metastasis does not necessarily confer a worse intrinsic prognosis, failure to recognize it may contribute to postoperative recurrence. Tang et al^[21]^ described solitary skip nodes located superior to the pancreatic neck, illustrating anatomically plausible variations in drainage. Therefore, for proximal or upper-body tumors, careful assessment of distal nodes is warranted even when regional metastasis is not apparent on imaging.

### 4.5. Metastatic patterns and prognosis

In our study, patients with disseminated lymphatic spread experienced markedly higher recurrence rates than those with localized spread (44.4% vs 18.2%), and their 3-year DFS and OS were significantly lower. Liu et al^[22]^ similarly highlighted the prognostic importance of metastatic extent and LNR. Li et al^[6]^ showed that lymph node sorting increases the detection of metastasis and improves staging accuracy. Our analysis also demonstrated a progressive rise in cross-regional involvement with advancing TNM stage, a trend consistent with previous reports.^[15]^ These results emphasize that metastatic distribution, differentiation grade, and LNR are central determinants of patient outcomes.

### 4.6. Implications for surgical strategy

These findings suggest that preoperative and intraoperative decision-making should account for tumor location, differentiation, and T stage. For patients with cardia tumors, advanced T stage, or poorly differentiated histology, a more extensive lymphadenectomy may be justified. Conversely, for distal, early-stage, or well-differentiated tumors, function-preserving approaches may be appropriate when oncologic safety is maintained. A case report by Zhu et al^[13]^ also highlighted nontraditional metastasis pathways – such as axillary nodal involvement – underscoring the variability of gastric lymphatic spread. Predictive nomograms and risk models have further enhanced individualized surgical planning and prognostic assessment.^[23]^

### 4.7. Limitations and future directions

Several sources of potential bias should be considered when interpreting the findings. First, as a retrospective single-center study, selection bias cannot be fully excluded. Patients undergoing curative gastrectomy in a tertiary hospital may represent a relatively selected population with better surgical eligibility, which could potentially underestimate the overall metastasis burden observed in the broader gastric cancer population. Second, heterogeneity in disease stage distribution might influence the observed association between tumor location and nodal involvement. Third, incomplete follow-up in a subset of patients may introduce survival estimation bias. Therefore, the magnitude and direction of these biases should be considered when interpreting the statistical associations observed in this study.

Another important limitation relates to potential structural bias associated with surgical technique. Although all patients underwent curative gastrectomy with systematic lymphadenectomy (D1+ or D2), variations in surgical approach – including open, laparoscopic, or robotic procedures – as well as differences in operative strategy (distal, proximal, or total gastrectomy) may influence the number and distribution of retrieved lymph nodes. Such variations could potentially affect the detection of metastatic stations and the calculated LNR. Although these factors were partially accounted for in the multivariate analysis, residual confounding cannot be completely excluded. Future multicenter prospective studies with standardized surgical protocols are needed to minimize this type of structural bias.

## 5. Conclusion

In summary, primary tumor location is strongly associated with lymphatic metastatic patterns in gastric cancer. Cardia tumors exhibit greater involvement of proximal and longitudinal nodal stations, whereas higher T stage, poor differentiation, and elevated LNR are linked with broader nodal spread and poorer survival. Incorporating primary tumor site into preoperative evaluation, alongside LNR and TNM staging, may substantially enhance risk assessment and guide individualized surgical planning.

## Author contributions

**Conceptualization:** Zhiqiang Dong, Liping Fu, Long Yang, Benxin Yang, Shuli Song, Jiangqiao Zhao.

**Data curation:** Zhiqiang Dong, Liping Fu, Long Yang, Benxin Yang, Shuli Song, Jiangqiao Zhao.

**Formal analysis:** Zhiqiang Dong, Liping Fu, Long Yang, Benxin Yang, Shuli Song, Jiangqiao Zhao.

**Funding acquisition:** Zhiqiang Dong, Liping Fu, Jiangqiao Zhao.

**Investigation:** Zhiqiang Dong, Jiangqiao Zhao.

**Writing – original draft:** Benxin Yang, Jiangqiao Zhao.

**Writing – review & editing:** Benxin Yang, Jiangqiao Zhao.
